# Information sampling differences supporting superior face identity processing ability

**DOI:** 10.3758/s13423-024-02579-0

**Published:** 2024-09-23

**Authors:** James D. Dunn, Sebastien Miellet, David White

**Affiliations:** 1 School of Psychology, UNSW Sydney, Kensington, NSW 2052 Australia; 2 School of Psychology, University of Wollongong, Wollongong, Australia

**Keywords:** Face perception, Facial recognition, Super-recognisers, Holistic processing, Individual differences, Forensic facial examiners, Forensic science

## Abstract

Face recognition in humans is often cited as a model example of perceptual expertise that is characterized by an increased tendency to process faces as holistic percepts. However emerging evidence across different domains of expertise points to a critical role of feature-based processing strategies during the initial encoding of information. Here, we examined the eye-movement patterns of super-recognisers—individuals with extremely high face identification ability compared with the average person—using gaze-contingent “spotlight” apertures that restrict visual face information in real time around their point of fixation. As an additional contrast, we also compared their performance with that of facial examiners—highly trained individuals whose superiority has been shown to rely heavily on featural processing. Super-recognisers and facial examiners showed equivalent face matching accuracy in both spotlight aperture and natural viewing conditions, suggesting that they were equally adept at using featural information for face identity processing. Further, both groups sampled more information across the face than controls. Together, these results show that the active exploration of facial features is an important determinant of face recognition ability that generalizes across different types of experts.

**Supplementary Information:**

The online version contains supplementary material available at 10.3758/s13423-024-02579-0.

People vary in how well they can recognise faces to a surprisingly large extent. Ability ranges from individuals with congenital prosopagnosia, who struggle to identify familiar faces and perform poorly on standardized tests (Duchaine & Nakayama, 2006), through to individuals known as “super-recognisers,” who possess extraordinary recognition skills in their everyday lives and achieve top scores on standardized tests (for comprehensive reviews, refer to Noyes et al., 2017; Ramon, 2021; also see Russell et al., 2009). These individual differences impact people’s daily lives and also have important applied implications in forensic and security settings.

Understanding the cognitive and perceptual processes driving differences in face processing ability can help to reveal the nature of perceptual expertise more broadly. For example, the finding that faces are processed more holistically than other objects (for reviews, see McKone & Yovel, 2009; Rossion, 2008; Tanaka & Simonyi, 2016) has also been found in people with expertise in other visual domains, including car recognition (Bukach et al., 2006), fingerprints (Busey & Vanderkolk, 2005; Vogelsang et al., 2017), medicine (Brams et al., 2019; Ivy et al., 2023; Sheridan & Reingold, 2017), chess (Brams et al., 2019), and elite sports (Mann et al., 2007). This appears to show that holistic processing is a key determinant of high performance on visual tasks.

However, evidence for the contribution of holistic processing to individual differences in people’s face identity processing ability has been mixed. Some studies have shown a weak association between interindividual differences in face processing ability and the extent to which they use “holistic” processing (DeGutis et al., 2013; Wang et al., 2012), but other studies have shown no association (Dunn et al., 2022; Konar et al., 2010; Rezlescu et al., 2017; Richler et al., 2015; Sunday et al., 2017; Tsantani et al., 2020; Ventura et al., 2022). This suggests that holistic processing may not be as fundamental to individual’s face recognition ability as might be inferred from previous work based on group-level analysis of performance differences across different stimulus classes (White & Burton, 2022).

Indeed, there is accumulating evidence that individual differences in face identity processing ability are predicted by feature-level processing during initial perceptual encoding. People with better face identity processing ability appear to be able to recognize faces with less visual information available (Dunn et al., 2022; Leong et al., 2023; Royer et al., 2015), exploit featural information more effectively (Tardif et al., 2019) and consistently (Nador et al., 2021) than typical viewers. Critically, the relationship between ability and these information sampling behaviours is strongest during the learning phase when participants first encounter and extract information from the face rather than when they later recognise the face (Dunn et al., 2022). This suggests that superior ability is linked to how faces are encoded into memory. Further, evidence from eye movements suggests differing strategies between encoding and recognition of faces, with the initial encoding of faces into memory being reliant on feature information gradually integrated over many fixations (Arizpe et al., 2019).

Recently, Dunn et al. (2022) examined the information sampling that supports super-recognisers superior face identity processing ability. To do this, they used a gaze-contingent spotlight task that modified the amount of information available to observers in real time. This paradigm used different-sized “spotlights” that revealed information around the centre of their eye gaze while they learnt and recognised faces to isolate the global and local sampling behaviours that support individual differences in face identity processing ability. They found that super-recognisers were more accurate at recognising faces than typical viewers across all spotlight sizes and explored the faces more than typical viewers, suggesting that they have superior featural processing and that their advantage is not contingent on holistic processing. Critically, accuracy was predicted more by information sampled during encoding than recognition. This indicates that information sampling is not simply mirrored between encoding and recognition (i.e., scan path replay hypothesis; Just & Carpenter, 1976), but rather the initial encoding of featural information is a distinct, and potentially fundamental, facet of ability (see also Arizpe et al., 2019).

Here, we investigated the role of encoding featural information for superior face identity processing using a face matching task. Face matching tasks require perceptual processing of faces with little reliance on memory, allowing us to focus on initial identity encoding rather than memory retention or recognition stages. To isolate the contribution of featural information, we delivered an adapted gaze-contingent “spotlight” paradigm to super-recognisers and compared their performance and sampling strategies when faces are visible in full view to when they are visible only through a spotlight aperture controlled by their eye movements. Using this approach, we assessed whether super-recognisers’ superior accuracy persists when forced to perform featural sampling.

We also aimed to differentiate between natural ability in recognising faces and aptitude in matching identity of face images that have been acquired through professional practice. To do this we recruited a small group of forensic facial examiners who perform matching decisions as part of their daily work (for review on facial examiners, see White et al., 2021). Comparing super-recognisers with forensic examiners enabled us to test for differences in their processing strategies for the first time. Prior work has shown equivalent accuracy in these groups for face matching tasks in which the whole face is visible (e.g., Dunn et al., 2023; Towler et al., 2023), but this could mask important underlying differences in processing strategy. Forensic facial examiners are known to rely more on feature-by-feature comparisons to make face identification decisions than do typical viewers (Claydon et al., 2022; Towler et al., 2019; White et al., 2021), but it is not clear whether they are also distinguishable from super-recognisers or whether these groups converge on similar strategies to attain their high levels of accuracy. If super-recognisers are more reliant on holistic processing than examiners, then they will show poorer accuracy than examiners when viewing faces through spotlight apertures (Towler et al., 2023).

## Method

### Participants

Forty super-recognisers (15 women, 25 men, *M*_age_ = 38.0 years, *SD*_age_ = 7.1) were recruited to participate in the study by an email advertisement sent to a database of volunteers who exceeded normal range on three standardised face identification tests administered online: the Cambridge Face Memory Test–Long Form (CFMT + ; Russell et al., 2009), the Glasgow Face Matching Test–Short Form (GFMT; Burton et al., 2010), and the UNSW Face Test (Dunn et al., 2020; for a description of the screening tests, see the Supplemental Material available online). All super-recognisers scored a mean *z* score above 1.7 standard deviations across these tests. This is a strict criterion relative to selection thresholds used in this field, and the requirement to show repeated high performance over subsequent tests limits any influence of chance or transient factors on performance (see Ramon, 2021).

Three forensic facial examiners were recruited from the Australian Passport Office (one woman, two men, *M*_age_ = 36 years, *SD*_age_ = 2.6). Examiners’ experience in the role ranged from 19 months to 5.5 years and all had completed multiple sessions of training in forensic facial comparison as well as on-the-job training and mentorship.

Thirty-five student controls (24 women, 10 men, one nonbinary, *M*_age_ = 19.2 years, *SD*_age_ = 1.8) volunteered to participate in exchange for course credit. All participants had normal or corrected-to-normal vision.

### Apparatus

Participants’ eye movements were recorded with the Tobii Pro Spectrum (with chin rest) or the EyeLink Portable Duo (with chin rest only) using the Eye-Link Toolbox (Cornelissen et al., 2002). These trackers have an average gaze-position error of about 0.25° and a spatial resolution of 0.01°. Only the dominant eye was tracked. The experiment was coded in MATLAB (The MathWorks Inc., 2022) using the Psychophysics Toolbox (Brainard, 1997). Calibrations for eye fixations were conducted at the beginning of the experiment using a nine-point fixation procedure using MATLAB software and repeated until the optimal calibration criterion was reached.

### Stimuli

**Fig. 1:**
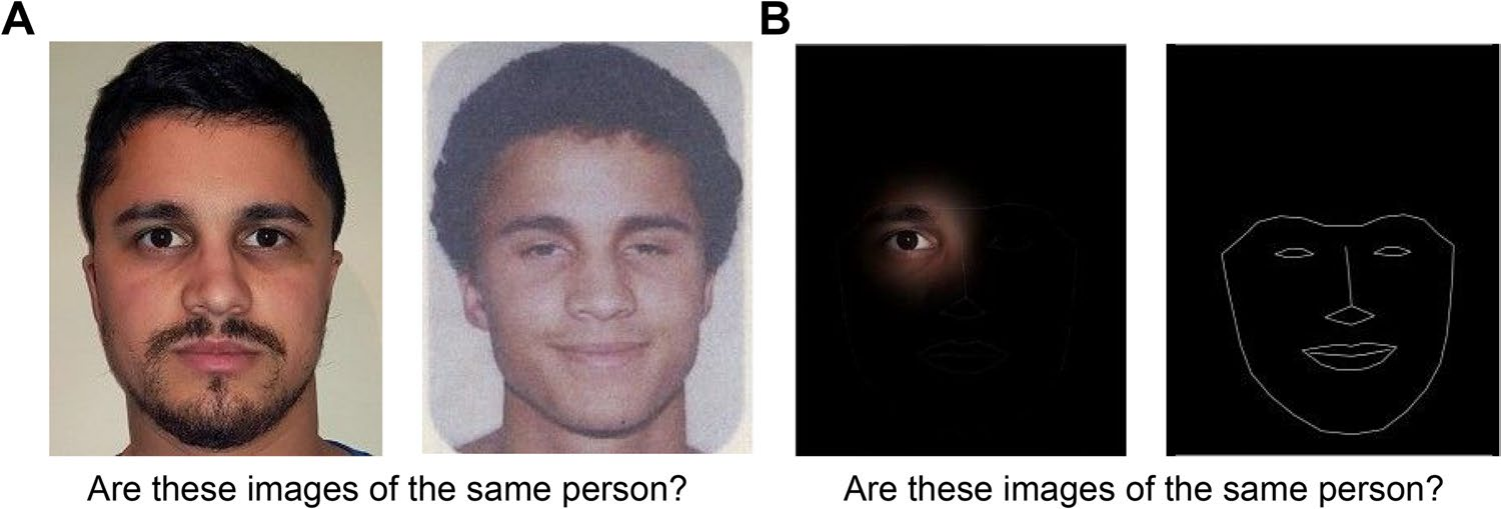
Example passport images for natural view (**A**) and spotlight (**B**) conditions. In the spotlight condition, the face information was only revealed at the location of participants’ gaze, with the rest of the face obscured by mask with a stencil outline of the facial features shown parafoveally for saccade programming. A video showing a screen recording during spotlight viewing is available (https://osf.io/mj6bq/), but note that the actual stimulus depends on moment-by-moment gaze position of the participants, and so it is not possible to illustrate the stimulus directly. Images are representative of the stimuli used in the matching task, but for reasons of privacy, we are not able to provide examples of the passport images used in our studies. Example images are of the same person

Stimuli used in this study were adult Australian passport images sampled from a database containing passport application images of 20,000 anonymous identities (White et al., 2015). The database was a random sample of consenting Australian citizens, with the demographic composition reflective of Australian demographics. For each identity, a distractor identity was selected as the most similar match by proprietary face recognition software from the passport database. For match images, the two passport images were taken about 10 years apart. Images were resized so that faces were approximately the same size and positioned so that the eye positions of the faces were aligned and presented side by side on the screen with 400 pixels between the centres of the two images (for an example, see Fig. 1A).

Spotlight aperture viewing was created by dynamically updating the image, revealing only visual information centred around the observer’s point of fixation (see Fig. 1B; for video demonstration, see https://osf.io/mj6bq/). Our previous studies demonstrated that this aperture disrupts global sampling and impacts visual exploration and performance (Dunn et al., 2022; Papinutto et al., 2017). For each face image, we created an image mask to cover the faces on spotlight trials that would also allow participants to orientate their gaze towards features. So that participants could program natural saccades within the fixated face image and between the two images, we created a stencilled outline of facial features on the masks. To create the stencil feature outline, we used automated face detection software (Dlib; King, 2009) to detect 68 fiducial landmarks within each face. We then plotted and drew connecting lines between related feature points to create a stencil outline of the position of each facial feature in the image.

The luminosity of spotlight information revealed was masked by a black image which progressively decreased the clarity of the information with greater pixel distance from the centre of the fixation, following a Gaussian function. We used an aperture size of 10° of visual angle, which corresponded to 24% preserved face information at each fixation (see also Dunn et al., 2022), as was the minimum size required to see a single whole feature within the face. We used progressive masking to avoid unnaturally attracting participants’ attention towards visual artefacts created by a hard aperture border.

### Procedure

Participants were shown two images simultaneously and asked whether they depicted the same person or two different people (Fig. 1). In half of trials, participants could see the full original face images for the duration of the trial (natural view). In the other half of trials, participants viewed the faces through the spotlight viewing apertures.

Images were shown for a maximum of 20 s before disappearing from the screen, and participants could make a decision before or after the images disappeared. Participants first completed two practice trials, followed by four blocks of 12 trials (48 trials in total) which were completed in a randomized order. Practice trials were not included in the final analysis.

#### Eye-movement classification and cleaning parameters

Fixations were coded with a threshold of 50° of visual angle per second; adjacent samples that were below the velocity threshold were coded as a single fixation, and samples above this threshold were coded as saccades. Fixation data were then cleaned using the following parameters: (a) Fixations within 0.5° of visual angle and 75 ms were merged, (b) fixations shorter than 50 ms were removed, and (c) fixations longer than three standard deviations from the mean fixation duration of each participant were removed to prevent biases caused by outlier or nongenuine fixations (Berger & Kiefer, 2021; Godwin et al., 2021). For trial-level analyses, trials were removed if there were no valid fixations.

We analysed information sampling patterns in pixel space using a linear mixed model in the iMap4 toolbox (Lao et al., 2017). Statistical fixation maps were created at the trial level for fixations within the images and normalized to account for differences between trials. To reduce the computational time, we down-sampled the fixation map to 76 × 56 pixels and applied a mask to only model the pixels with an average duration larger than 33 ms. Fixation duration maps were first smoothed at 2º of visual angle.

## Results

### Accuracy

**Fig. 2:**
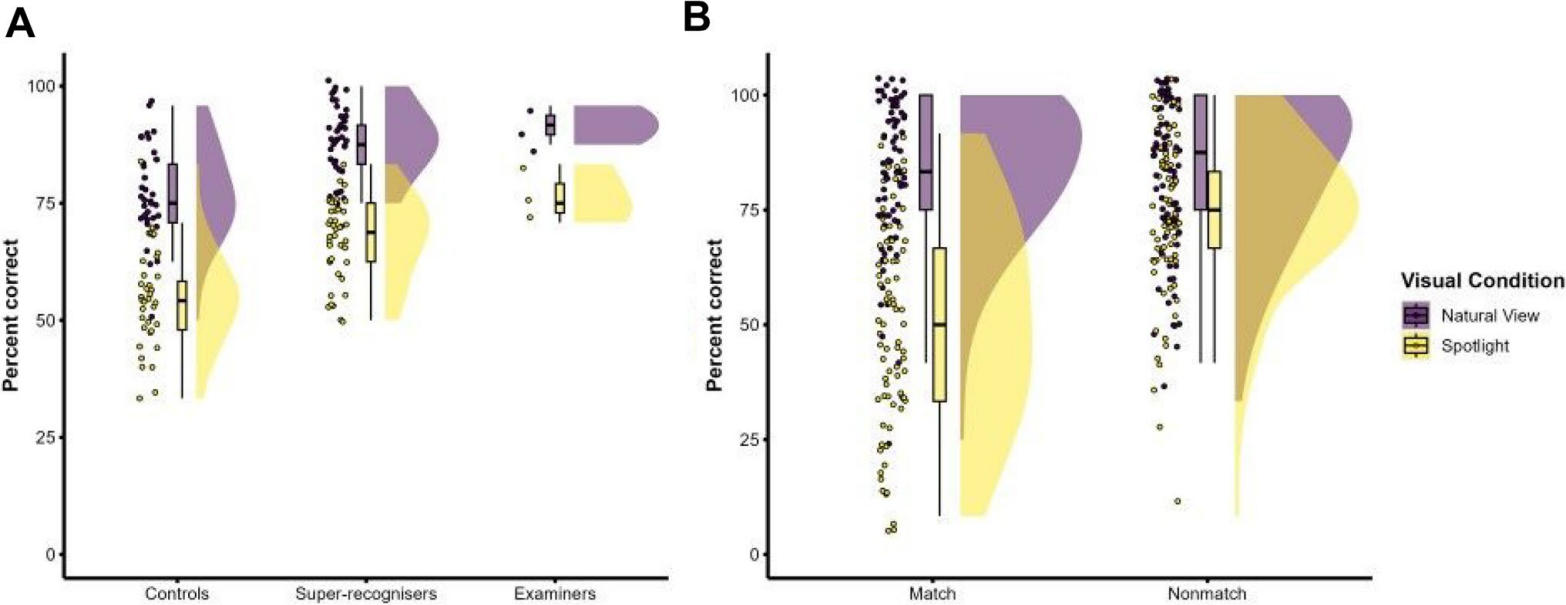
Raincloud plots showing significant main effects and interactions in accuracy data. **A** Main effects show that super-recognisers and examiners both were more accurate than controls but were not significantly different from each other. Further, matching faces with the spotlight aperture made all groups less accurate by similar degrees. **B** Interaction shows spotlight reduces accuracy more on match trials than nonmatch trials. (Colour figure online)

The percentage of correct responses was analysed in a 3 × (2 × 2) mixed-factors analysis of variance (ANOVA), with group (controls, super-recognisers, examiners) as the between-subjects factor and visual condition (natural view, spotlight) and trial type (match, nonmatch) as the within-subjects factors (see Fig. 2). Follow-up comparisons were performed using Welch’s unequal variances *t* test as it is more reliable when the two samples have unequal variances and unequal sample sizes (Delacre et al., 2017).

There was a significant main effect of group, *F*(2, 75) = 37.36, *p* < 0.001, η_p_^2^ = 0.50. Follow-up comparisons reveal significantly higher accuracy for super-recognisers and examiners compared with controls, super-recognisers vs control: *t*(70.69) = 7.99, *p* < 0.001, Cohen’s *d* = 1.85; examiner vs control: *t*(2.90) = 6.66, *p* = 0.008, Cohen’s *d* = 2.77. However, there was no significant difference between super-recognisers and examiners, *t*(2.71) = 2.28, *p* = 0.116, Cohen’s *d* = 0.97.

There was a significant main effect of visual condition, with higher accuracy in the natural view than spotlight conditions, *F*(1, 75) = 60.81, *p* < 0.001, η_p_^2^ = 0.45. There was also a significant main effect of trial type, with higher accuracy for nonmatch than for match trials, *F*(1, 75) = 5.57, *p* = 0.021, η_p_^2^ = 0.07. These two main effects were modulated by a significant two-way interaction between visual condition and trial type factors, *F*(1, 75) = 8.11, *p* = 0.006, η_p_^2^ = 0.10. Follow-up comparisons reveal this interaction was driven by a larger reduction in accuracy for the spotlight condition from natural view for match trials, *t*(77) = 14.02, *p* < 0.001, Cohen’s *d* = 1.74, than for nonmatch trials, *t*(77) = 5.13, *p* < 0.001, Cohen’s *d* = 0.57.

The three-way interaction and two-way interactions between group and the other factors were not significant, *F* < 1.9, *p* > 0.16, signalling that the impairment caused by aperture viewing was equivalent across participant groups.

#### Individual differences in accuracy for natural view and spotlight

We explored the relationship between featural processing and face matching ability more closely by asking whether performance in natural viewing was predicted by accuracy in spotlight viewing. This would suggest that piecemeal comparison is used in unfamiliar face matching tasks, as has been proposed in prior work (Megreya & Burton, 2006).

**Fig. 3:**
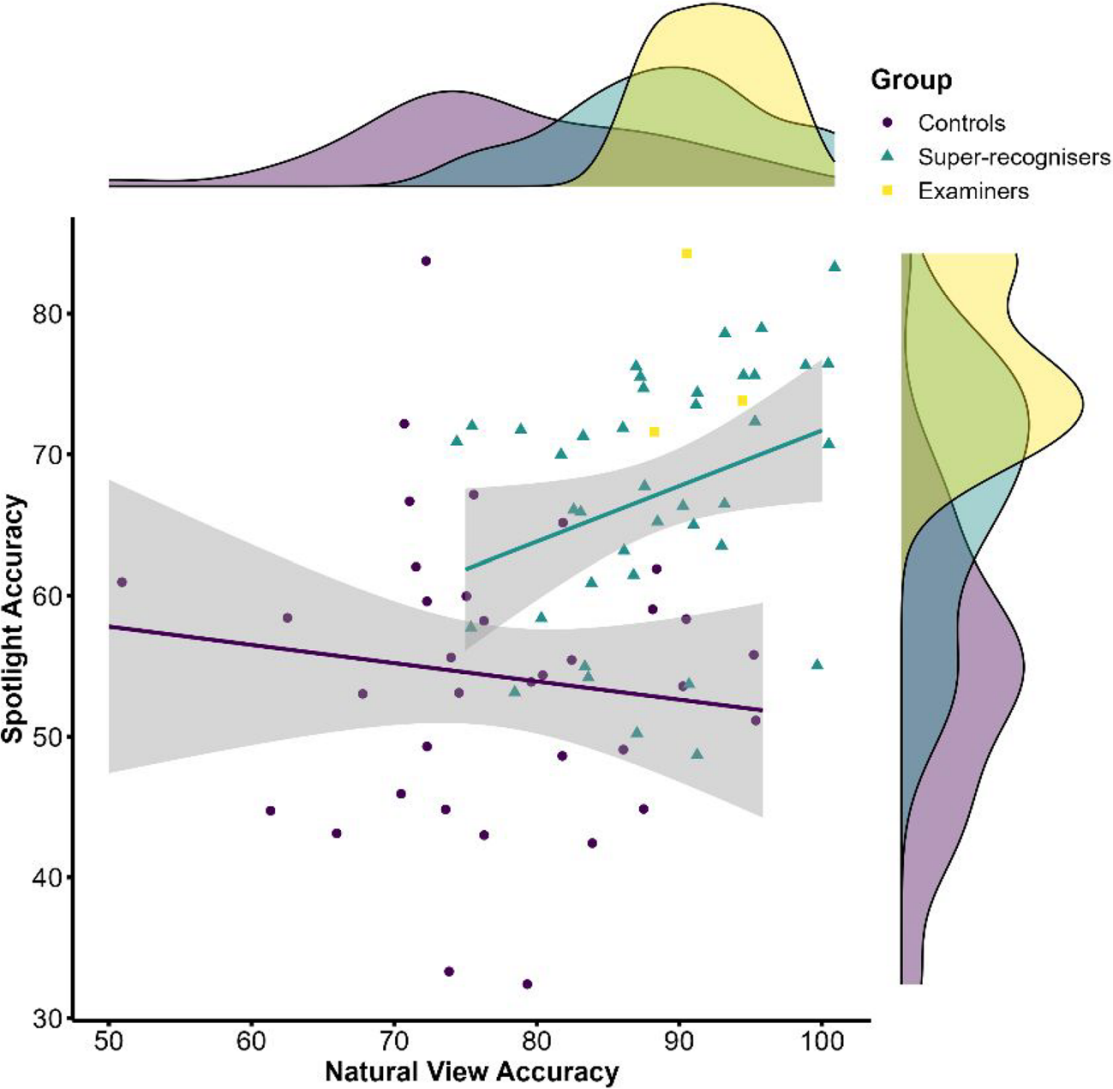
Scatterplot showing the relationship between accuracy between natural view and spotlight trials for each group. Analysis shows positive relationship between natural view and spotlight trials for super-recognisers but not controls. (Colour figure online)

The association between participants' accuracy on spotlight trials and natural view trials is shown in Fig. 3 with Spearman’s rank-order correlation showing a positive association, *r*_*s*_(76) = 0.40, *p* < 0.001. However, examining this relationship separately in each group reveals that this correlation only emerged for super-recognisers, *r*_*s*_(40) = 0.38, *p* = 0.015, and not controls, *r*_*s*_(35) =  − 0.14, *p* < 0.43 (see Fig. 3). This discrepancy between super-recognisers and controls may be attributed to the near chance-level accuracy observed in controls on spotlight trials—however, a one-sample *t* test shows control accuracy on spotlight trials is above chance: *t*(34) = 2.47, *p* = 0.019. Despite the positive relationship found for super-recognisers, visual inspection of Fig. 3 also reveals substantial interindividual differences in the effect of spotlight performance on accuracy of individual super-recognisers. Some super-recognisers appear relatively unaffected by spotlight viewing, whereas a significant proportion (*n* = 7) have their accuracy reduced to chance levels. Therefore, the overall group performance in super-recognisers might mask substantial heterogeneity in the processing strategies used by individual super-recognizers.

### Response latency

Response latency was analysed in a 3 × (2) mixed-factors ANOVA, with group (controls, super-recognisers, examiners) as the between-subjects factor and visual condition (natural view, spotlight) as the within-subjects factor.

There was a significant main effect of group, *F*(2, 75) = 13.62, *p* < 0.001, η_p_^2^ = 0.27. Follow-up comparisons show significantly longer response latencies for super-recognisers and examiners compared with controls, super-recogniser vs control: *t*(62.5) = 5.00, *p* < 0.001, Cohen’s *d* = 1.18; examiner vs control: *t*(4.03) = 3.23, *p* = 0.032, Cohen’s *d* = 1.02. There was no significant difference in response latencies between super-recognisers and examiners, *t*(2.92) = 0.11, *p* = 0.919, Cohen’s *d* = 0.04. There was a significant main effect of visual condition, *F*(1, 75) = 86.7, *p* < 0.001, η_p_^2^ = 0.54, as response latency was on average 5.2 s slower on spotlight trials than natural view trials.

These effects were moderated by a significant interaction between group and visual condition, *F*(2, 75) = 3.5, *p* = 0.035, η_p_^2^ = 0.09. Simple main effects show this interaction was caused by the a larger increase in response latency for spotlight compared with natural view for the examiners, mean difference = 10.4 s, *t*(2) = 8.21, *p* = 0.015, Cohen’s *d* = 4.46, than controls, mean difference = 4.8 s, *t*(34) = 8.93, *p* < 0.001, Cohen’s *d* = 0.79, and super-recognisers, mean difference = 5.3 s, *t*(39) = 8.66, *p* < 0.001, Cohen’s *d* = 1.38.

### Information sampling

**Fig. 4:**
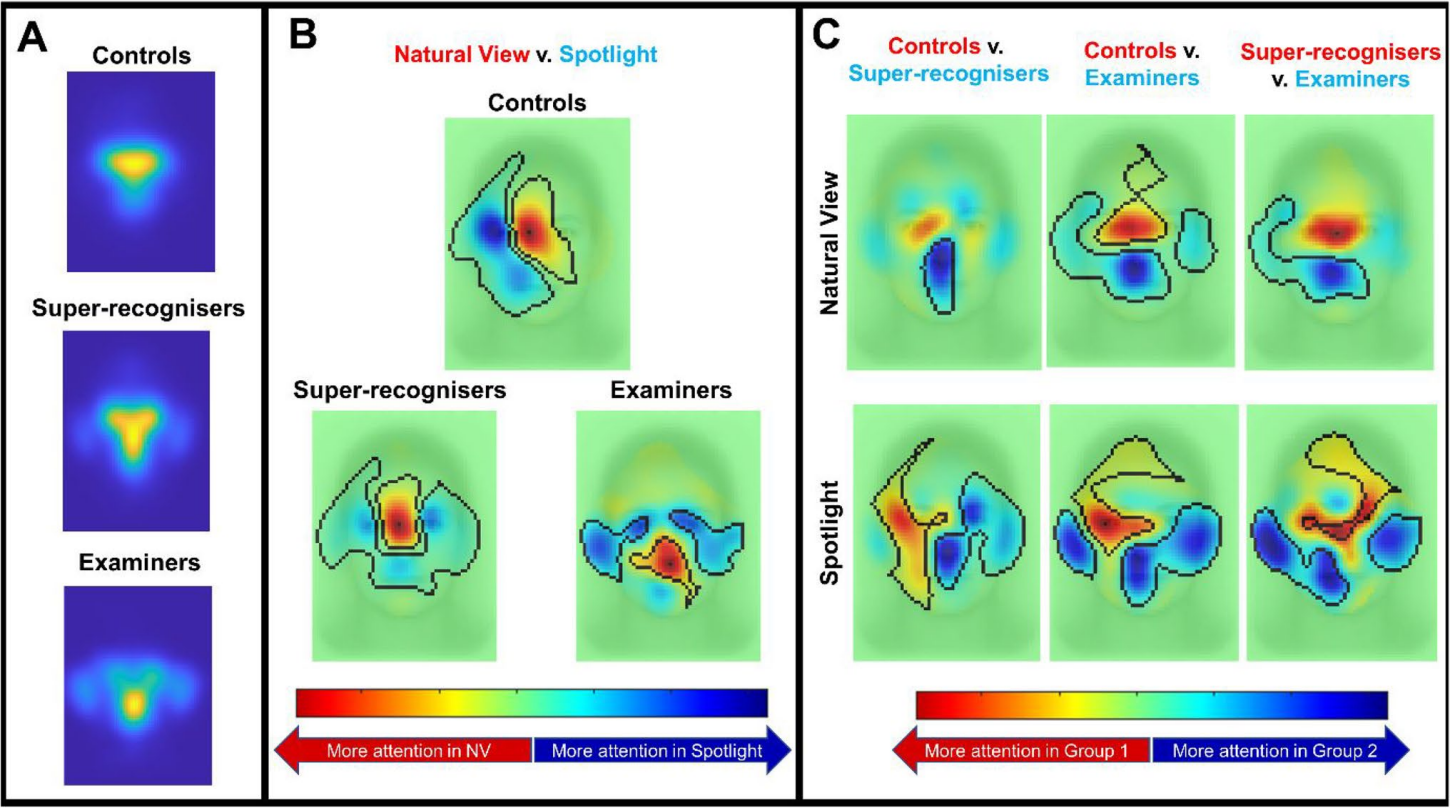
Heatmaps and statistical maps showing areas of statistical difference in gaze patterns in key contrasts.** A** Heatmaps show the average gaze patterns for each group. **B** These maps reveal a shift in information sampled between natural view and spotlight trials for all groups. **C** Comparisons between groups highlight the differences in information sampled separately for natural view (top row) and spotlight (bottom row) trials. (Colour figure online)

We analysed information sampling patterns using a linear mixed model in the iMap4 toolbox (Lao et al., 2017).^1^ All main effects and simple effects were tested for significance using a robust statistical approach of correcting for multiple comparisons by using bootstrap clustering (see Lao et al., 2017, for more details). This analysis showed a significant interaction between group and visual condition, which is visualised as a heatmap in Fig. 4.

Visual inspection of Fig. 4 reveals the nature of the interaction. Figure 4A shows that all groups showed a shift in information sampled between natural view and spotlight trials. In general, there is a shift from more central attention towards more peripheral regions of the face in the spotlight aperture viewing, but the qualitative nature and degree of this shift differed subtly between groups. This pattern is likely to reflect the fact that participants are not able to perceive peripheral features with more central fixations during aperture viewing.

Figure 4B shows the interaction broken down by simple main effects of group at each level of viewing condition. Group differences in viewing patterns are similar in both natural and spotlight conditions, but these differences are slightly more pronounced in the spotlight condition. In general, controls tend to fixate the eye region more than both super-recognisers and examiners, whereas both super-recognisers and examiners disperse their attention more broadly across other regions of the face. It also appears that examiners disperse their attention more broadly than did super-recognisers.

To assess whether group differences emerge in the early stages of information sampling, we examined the information sampled during the first three fixations using the same linear mixed modelling described above. Controls and super-recognisers exhibited similar fixation patterns during this period, contrasting with examiners, who focused significantly less on the eyes compared with both groups. This implies that distinctions in Examiner gaze behaviour arise from the earliest fixations on the face, likely a result of their training and examination protocol, while disparities between controls and super-recognisers only become evident in later information sampling. Full analysis of the first three fixations is available in the Supplementary Material.

#### Visual exploration

Analysis of information sampling suggests that there were group differences in the degree of visual exploration, with super-recognisers and examiners showing less anchoring on the eye region when compared with controls. To quantify this, we compared each group’s dispersal of gaze using the Gini coefficient, a statistical measure of dispersion (Lorenz, 1905), using the Gini coefficient and the Lorentz curve toolbox (Lengwiler, 2010). When gaze maps are analysed, higher Gini coefficients index a higher concentration of fixations on focal regions, whereas lower Gini coefficients index a greater dispersal of fixations across the face. This measure has been previously used to show that super-recognisers have greater visual exploration of faces than typical viewers both when learning and recognizing faces in a face memory paradigm (Dunn et al., 2022).

**Fig. 5:**
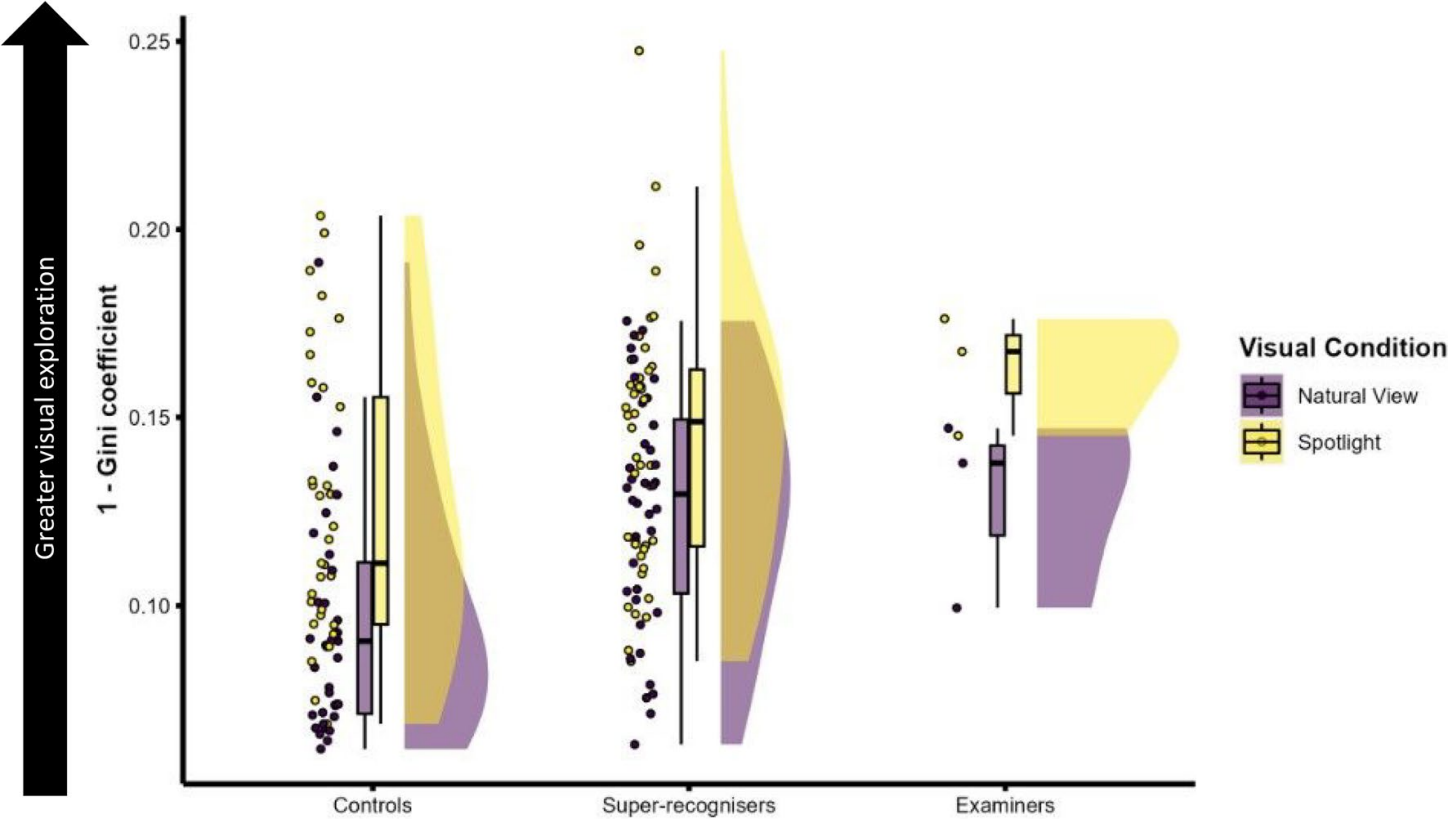
Raincloud plot showing group-level differences in visual exploration as measured by the Gini coefficient. The *y*-axis shows 1 minus Gini coefficient for ease of interpretation, as lower raw Gini scores indicate more exploration. Importantly, super-recognisers and examiners explored faces more than controls in natural view, independently of the time taken to study faces (see main text for details). (Colour figure online)

Distributions of Gini coefficient scores for each group in each condition are shown in Fig. 5. Visual inspection shows a clear effect of group, with super-recognisers and examiners exploring face images more than controls. All groups also appear to explore images more in the spotlight condition, thereby corroborating the pattern of results in the information sampling analysis above.

To test these observations, we analysed Gini coefficient scores at the trial level using a linear mixed model with the GAMLj module package in Jamovi (The Jamovi Project, 2022), with group and visual condition as fixed factors, and participant and image intercept as random effects. Because the task was self-paced it was also important to account for differences in response latency between groups, so we also included response latency as a covariate.^2^

We found response latency was a significant predictor of visual exploration, *F*(1, 3567.3) = 1283.23, *p* < 0.001, with larger response times associated with more visual exploration. Controlling for differences in response latency, there was a significant interaction between group and visual condition, *F*(2, 3451.3) = 14.83, *p* < 0.001, reflecting group differences in exploration for the natural view but not spotlight condition. Simple effects show this interaction was caused by significantly more exploration in super-recognisers than controls for natural view, *b* =  − 0.016, CI_95_ [− 0.026, − 0.005], *t*(83) =  − 3.09, *p* = 0.003, but not spotlight, *b* =  − 0.004, CI_95_ [− 0.014, 0.006], *t*(83.2) = 0.74, *p* = 0.462. The difference between examiners and controls was also significant for natural view, *b* =  − 0.026, CI_95_ [− 0.052, 0.001], *t*(81.3) =  − 2.01, *p* = 0.015, but not spotlight, *b* =  − 0.016, CI_95_ [− 0.042, 0.010], *t*(81.7) =  − 1.23, *p* = 0.221. There was again no difference between Super-recognisers and examiners in visual exploration in either condition, natural view: *b* = 0.01, CI_95_ [− 0.015, − 0.037], *t*(81.4) = 0.82, *p* = 0.415, spotlight: *b* = 0.012, CI_95_ [− 0.014, 0.038], *t*(81.3) = 0.95, *p* = 0.343.

Together, these results show that super-recognisers and examiners both explored the face more than controls in natural viewing conditions, even when accounting for differences in how long they spent sampling the faces.

## Discussion

Our study investigated the contribution of facial-feature processing towards individual differences in face identity processing ability. We compared super-recognisers—people with outstanding natural ability in face recognition—to both typical viewers and forensic facial examiners that have received extensive professional training in facial-image comparison. Consistent with a recent study employing a recognition memory paradigm (Dunn et al., 2022), we found that super-recognisers outperformed controls at a similar level regardless of whether they had a full view of the face or could only sample small regions of the face on each fixation. Super-recognisers also performed with comparable accuracy to forensic examiners even in the aperture viewing condition. Prior work has shown that forensic examiners use a distinct feature-based processing approach to comparing face images that distinguishes them from standard participant groups (Towler et al., 2017; White et al., 2015). Our study shows that super-recognisers are also highly adept at feature-based identity processing.

Visual exploration and feature processing were common denominators of high performance across different types of high-performing groups. We found that super-recognisers’ and forensic examiners’ visual exploration was more spatially distributed across the face compared with controls, thus supporting the sampling of a greater number of features. This was corroborated by the analysis of Gini coefficients showing higher levels of gaze dispersal for super-recognisers and forensic examiners than controls. It is important to note that the simultaneous face matching task we used here is purely perceptual in the sense that perceptual access is available to both images until the participant is ready to make a response.

In this context, our findings provide evidence that differences in face identity processing between super-recognisers and typical viewers emerge from perceptual encoding of face identity. Thus, the present findings are in line with prior work showing that super-recognisers sample a greater volume of face information during the learning phase of a face recognition task (Dunn et al., 2022). This greater visual exploration may support super-recognisers superior feature encoding by facilitating the discovery of identity-specific diagnostic facial features (Dunn et al., in press). Indeed, super-recognisers and forensic examiners fixated features which have been linked to more accurate recognition (e.g., eyes; Royer et al., 2018; Towler et al., 2017) over other features which are less diagnostic (e.g., mouth; Bukach et al., 2008; Caldara et al., 2005).

Together with other work showing that people with high levels of face recognition ability are adept at processing identity from isolated facial regions (Leong et al., 2023; Royer et al., 2015; Tardif et al., 2019), our results provide an important constraint on the theoretical standpoint that holistic processing underpins individual differences in face recognition ability (e.g., DeGutis et al., 2013). It remains possible that the ability to construct a holistic representation of the face can also stem from active processing of discrete samples of local information (e.g., see Avidan & Behrmann, 2021), but this conceptualisation of holistic processing is very different to the traditional view that holistic perception can be inferred from global sampling around the centre of the face (Bombari et al., 2009).

It is also worth noting that there was substantial variability within the super-recogniser group. First, there was substantial variability in the overall accuracy of individual super-recognisers on the face matching task. This is expected because test correlations between different face identity processing task formats is typically in the moderate range (*r* = 0.4 to 0.6), and some regression to the mean is expected in line with prior work (e.g., Dunn et al., 2023; Nador et al., 2022; Towler et al., 2023). However, there was also substantial variation in the extent of impairment caused by spotlight viewing in individual super-recognisers. Figure 3 shows that while most of the super-recognisers performed remarkably well in the spotlight condition, for some it caused drastic reductions in accuracy. This could point to cognitive heterogeneity in the processing mechanisms used by individual super-recognisers to perform the task. If future work were to find that this represents a stable individual difference, then it would suggest there are a variety of different routes to optimizing face identity processing ability.

Finally, although super-recognisers and examiners performed more similarly to each other than they did to control participants, there were also some subtle differences between them. Examiners directed attention more to external facial features and this was especially noticeable in the first three fixations they made, which were less likely to be directed to the eyes than for either super-recognisers or controls. This aligns with the fact that forensic face examiners receive training that encourages more systematic patterns of attention to highly diagnostic features such as the ears (Claydon et al., 2022; Towler et al., 2017, 2019). By comparison, super-recognisers appeared to act like controls during initial fixations to the face, before adopting a more exploratory process in later fixations. This might point to greater flexibility in super-recognisers gaze patterns compared with both controls and forensic examiners.

Overall, the findings of this study highlight the importance of the initial encoding of featural information for accurate superior face matching performance. Both super-recognisers and forensic facial examiners demonstrated remarkable accuracy even when their view of faces was limited. Along with the enhanced visual exploration exhibited by both groups, this implies a limited role for holistic perceptual encoding of faces in superior face identity processing ability.

## Supplementary Information

Below is the link to the electronic supplementary material.

Supplementary file1 (DOCX 1559 kb)

## Data Availability

The datasets generated during and analysed during the current study are available in the Open Science Framework repository, (https://osf.io/mj6bq/). Experimental materials cannot be shared due to privacy constraints on images.

## Footnotes

1 Full model terms: Pixel Intensity ~ 1 + Group × TrialType + Group × VisualCondition + TrialType × VisualCondition + Group:TrialType:VisualCondition + ( 1 | Subject) + (1 | Image).

2 Full model terms: Gini coefficient ~ 1 + Group + VisualCondition + Group × VisualCondition + Response Latency + ( 1 | Subject) + (1 | Image).

## Code availability

The analysis scripts are available (https://osf.io/mj6bq/). The iMAP4 package can be downloaded from https://au.mathworks.com/matlabcentral/fileexchange/51234-imap4

## Declarations

### Open practices statement

The data and analysis scripts are available online (https://osf.io/mj6bq/). The experiment was not preregistered.
