# Supplementary material for: Information sampling differences supporting superior face identity processing ability
Source: Psychon Bull Rev. 2024 Sep 23;32(2):801–11. doi: 10.3758/s13423-024-02579-0 (PMC12000253; doi:10.3758/s13423-024-02579-0)
Supplement: Supplementary file 1 — Supplementary file1 (DOCX 1559 kb) [file 13423_2024_2579_MOESM1_ESM.docx]

# Information sampling differences supporting superior face identity processing ability Supplementary Materials

James D. Dunn^1^, Sebastien Miellet^2^, David White^1^

^1^ School of Psychology, UNSW Sydney, Australia

^2^ School of Psychology, University of Wollongong, Australia

Corresponding Author: James D. Dunn,. Email: [j.d.dunn@unsw.edu.au](mailto:j.d.dunn@unsw.edu.au).

## Screening Tests

### Cambridge Face Memory Test Long Form (CFMT+)

The CFMT+ (Russell et al., 2009) assesses individuals' aptitude for acquiring and identifying unfamiliar faces. It involves the process of acquainting participants with specific target faces, which they must subsequently identify within three-choice selection scenarios. The examination comprises four progressive stages, each escalating the complexity of the task. These stages introduce untrained angles of view, alter lighting conditions in the images, incorporate visual distortions, and vary facial expressions across images. Normative statistics for this test were drawn from the study conducted by Bobak et al. (2016).

### Glasgow Face Matching Test Short Form (GFMT)

The GFMT (Burton et al., 2010) (Burton et al., 2010) serves as an assessment of individuals' proficiency in face matching. In the short version of the GFMT, participants are tasked with determining whether pairs of faces depict the same individual or two distinct individuals (comprising 20 matches and 20 non-matches). These images feature the same person, captured moments apart on the same day, but using two different cameras. Statistical norms for this examination were derived from the research conducted by Burton et al. (2010).

### UNSW Face Test

The UNSW Face Test (Dunn et al., 2020) is an assessment that evaluates individuals' overall face recognition skills, encompassing both face memory and face matching abilities. This test combines studio-captured images with ambient images sourced from social media platforms like Facebook for each target individual.

The UNSW Face Test consists of two sequential subtasks: a recognition memory task and a match-to-sample sorting task. In the recognition memory task, participants are presented with 20 faces, each displayed for 5 seconds. They are then tested on new images of these same 20 identities, mixed with 20 additional distractor faces.

In the match-to-sample task, participants are given 5 seconds to memorize a target face and must subsequently sort a set of four ambient images. They drag the correct image to the right if it matches the target face or to the left if it does not. Participants are informed that the image set could contain between 0 and 4 target images, with the remaining images in the set being foils. This subtask includes two practice trials followed by 20 trials in a predetermined sequence.

Normative data for this test was collected and reported in Dunn et al. (2020).

## Super-recogniser Screening Test Results

Supplementary Table 1 shows the recruited super-recognisers scores on each screening test.

**Supplementary Table 1.** Recruited super-recognisers scores on screening tests.

| Subject | Percent correct | | | Z score | | | |
| --- | --- | --- | --- | --- | --- | --- | --- |
|  | *GFMT* | *CFMT+* | *UNSW* | *GFMT* | *CFMT+* | *UNSW* | *Mean* |
| SR01 | 100 | 99 | 78 | 1.93 | 2.45 | 3.21 | 2.53 |
| SR02 | 100 | 85 | 83 | 1.93 | 1.37 | 4.21 | 2.5 |
| SR03 | 100 | 85 | 83 | 1.93 | 1.37 | 4.07 | 2.46 |
| SR04 | 100 | 89 | 79 | 1.93 | 1.72 | 3.49 | 2.38 |
| SR05 | 100 | 92 | 77 | 1.93 | 1.97 | 3.06 | 2.32 |
| SR06 | 100 | 90 | 78 | 1.93 | 1.8 | 3.21 | 2.31 |
| SR07 | 100 | 84 | 80 | 1.93 | 1.29 | 3.64 | 2.28 |
| SR08 | 100 | 96 | 73 | 1.93 | 2.31 | 2.34 | 2.2 |
| SR09 | 100 | 89 | 76 | 1.93 | 1.72 | 2.92 | 2.19 |
| SR10 | 95 | 78 | 84 | 1.41 | 0.77 | 4.36 | 2.18 |
| SR11 | 100 | 93 | 73 | 1.93 | 2.06 | 2.49 | 2.16 |
| SR12 | 95 | 89 | 78 | 1.41 | 1.72 | 3.35 | 2.16 |
| SR13 | 95 | 95 | 75 | 1.41 | 2.23 | 2.78 | 2.14 |
| SR14 | 95 | 93 | 76 | 1.41 | 2.06 | 2.92 | 2.13 |
| SR15 | 100 | 91 | 73 | 1.93 | 1.89 | 2.49 | 2.1 |
| SR16 | 100 | 95 | 71 | 1.93 | 2.23 | 2.06 | 2.07 |
| SR17 | 100 | 89 | 74 | 1.93 | 1.65 | 2.63 | 2.07 |
| SR18 | 100 | 87 | 74 | 1.93 | 1.54 | 2.63 | 2.03 |
| SR19 | 100 | 86 | 74 | 1.93 | 1.46 | 2.63 | 2.01 |
| SR20 | 100 | 88 | 73 | 1.93 | 1.63 | 2.34 | 1.97 |
| SR21 | 100 | 91 | 71 | 1.93 | 1.89 | 2.06 | 1.96 |
| SR22 | 100 | 86 | 73 | 1.93 | 1.46 | 2.49 | 1.96 |
| SR23 | 98 | 92 | 72 | 1.67 | 1.97 | 2.2 | 1.95 |
| SR24 | 100 | 92 | 70 | 1.93 | 1.97 | 1.91 | 1.94 |
| SR25 | 95 | 75 | 82 | 1.41 | 0.43 | 3.93 | 1.92 |
| SR26 | 100 | 89 | 71 | 1.93 | 1.72 | 2.06 | 1.9 |
| SR27 | 98 | 79 | 77 | 1.67 | 0.86 | 3.06 | 1.86 |
| SR28 | 100 | 88 | 70 | 1.93 | 1.63 | 1.91 | 1.82 |
| SR29 | 100 | 83 | 73 | 1.93 | 1.2 | 2.34 | 1.82 |
| SR30 | 98 | 80 | 75 | 1.67 | 0.95 | 2.78 | 1.8 |
| SR31 | 95 | 83 | 75 | 1.41 | 1.2 | 2.78 | 1.8 |
| SR32 | 93 | 72 | 83 | 1.15 | 0.17 | 4.07 | 1.8 |
| SR33 | 98 | 91 | 69 | 1.67 | 1.89 | 1.77 | 1.78 |
| SR34 | 98 | 81 | 74 | 1.67 | 1.03 | 2.63 | 1.78 |
| SR35 | 95 | 87 | 73 | 1.41 | 1.54 | 2.34 | 1.77 |
| SR36 | 90 | 86 | 76 | 0.9 | 1.46 | 2.92 | 1.76 |
| SR37 | 100 | 80 | 73 | 1.93 | 0.95 | 2.34 | 1.74 |
| SR38 | 95 | 89 | 71 | 1.41 | 1.72 | 2.06 | 1.73 |
| SR39 | 98 | 89 | 69 | 1.67 | 1.72 | 1.77 | 1.72 |
| SR40 | 98 | 89 | 69 | 1.67 | 1.72 | 1.77 | 1.72 |

The high cut-off for eligibility as a super-recogniser means that there is marginal variability in scores on the Glasgow Face Matching Test (GFMT) (range = 10%), and reduced variability on the Cambridge Face Memory Test (CFMT+) (range = 27%) and UNSW Face Test (range = 15%) compared to test norms. However, to determine whether these diagnostic tests predict task performance, we conducted a correlational analysis (Spearman’s rank order). This showed no significant correlations between CFMT and UNSW Face Test scores and super-recognisers' accuracy on natural view trials, spotlight trials, or overall performance (*r_s_*(40) < 0.29, *p* > .071).

## Ruling out ceiling effects in accuracy

To rule out ceiling effects impacting our interpretation of results, we conducted an additional Robust Paired Samples T-Test analysis to compare the effect of Spotlight separately for each group using the Walrus package for JAMOVI (Love & Mair, 2017). This approach trims the data to remove potential effects caused by the tails of the distribution and outliers (see also Robust Statistical Methods: Wilcox, 2012). This analysis showed similar-sized reductions in accuracy for all three groups, with the largest reduction found for Control participants (Mean difference = 22.6%, *t*(20) = 8.2, *p* < .001, Cohen’s *d* = 0.809), followed by Super-recognisers (Mean difference = 20.1%, *t*(23) = 11.9, *p* < .001, Cohen’s *d* = 0.941), then Examiners (Mean difference = 15.3%, *t*(2) = 4.16, *p* = .053, Cohen’s *d* = 0.907). This pattern of results would suggest that, if anything, Controls and not Super-recognisers rely more on holistic processing, which does not corroborate the idea that high performance is due to holistic processing.

## Analysis of first 3 fixations

To assess whether group differences emerge in the early stages of information sampling, we examined the information sampled during the first 3 fixations using Linear Mixed-Model in the iMap4 toolbox (Lao et al., 2017)^[[1]](#footnote-1)^.

Controls and Super-recognisers exhibited similar fixation patterns during this period, with no significant difference in gaze on Natural View trials and the only difference in Spotlight trials being a lateral distinction between sampling more of the left or right eye (Supplementary Figure 1). This is in contrast to Examiners, who focused significantly less on the eyes compared to both groups in both Natural View trials and Spotlight trials. The high-level of overlap in gaze by Controls and Super-recognisers suggests that in early information sampling these groups are equivalent, which means that any differences found between groups when looking at the gaze across the whole trial occurs after the first fixations. This is distinct from Examiners, who’s extensive training and protocol may have resulted in different information sampling strategies from untrained groups. While not statistically significant, there are suggestions that Examiners instead begin by looking more at the nose both with full view of face and under restricted viewing conditions, which is likely a result of their standardised examination protocol.


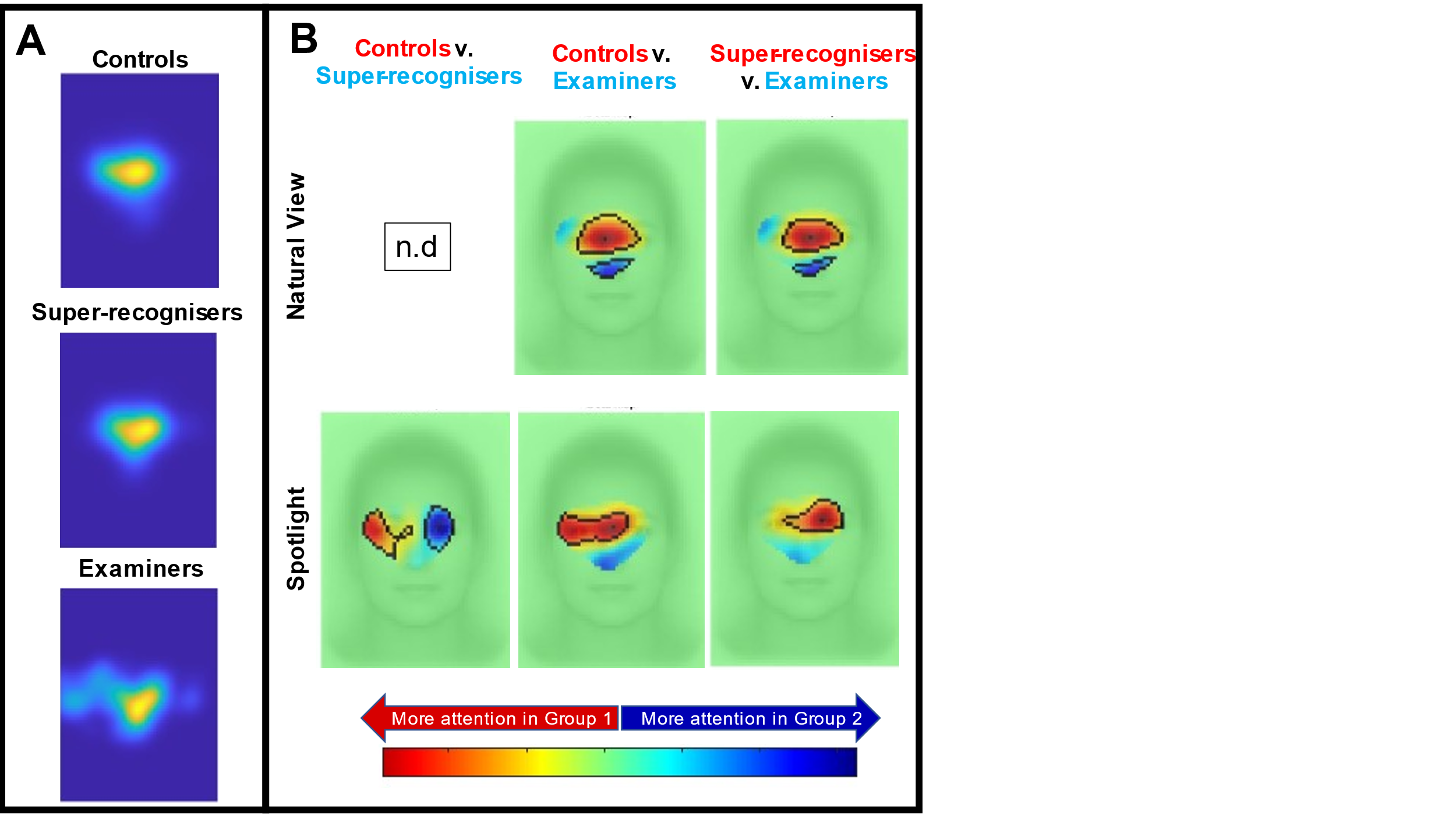


**Supplementary Figure 1.** Heatmaps and statistical maps showing areas of statistical difference in gaze patterns in key contrasts for the first three fixations. (A) Heatmaps showing the average gaze patterns for each group. (B) Comparisons between groups highlight the differences in information sampled in first three fixations separately for Natural View (top row) and Spotlight trials (bottom row).

References

Bobak, A. K., Pampoulov, P., & Bate, S. (2016). Detecting Superior Face Recognition Skills in a Large Sample of Young British Adults. *Front Psychol*, *7*, 1378. <https://doi.org/10.3389/fpsyg.2016.01378>

Burton, A. M., White, D., & McNeill, A. (2010). The Glasgow Face Matching Test. *Behav Res Methods*, *42*(1), 286-291. <https://doi.org/10.3758/BRM.42.1.286>

Dunn, J. D., Summersby, S., Towler, A., Davis, J. P., & White, D. (2020). UNSW Face Test: A screening tool for super-recognizers. *PLoS One*, *15*(11), e0241747. <https://doi.org/10.1371/journal.pone.0241747>

Russell, R., Duchaine, B., & Nakayama, K. (2009). Super-recognizers: people with extraordinary face recognition ability. *Psychon Bull Rev*, *16*(2), 252-257. <https://doi.org/10.3758/PBR.16.2.252>

Wilcox, R. R. (2012). *Introduction to robust estimation and hypothesis testing*. Academic press.

1. Full model terms: Pixel Intensity ~ 1 + Group*TrialType + Group*VisualCondition + TrialType*VisualCondition + Group:TrialType:VisualCondition + ( 1 | Subject) + (1 | Image) [↑](#footnote-ref-1)
